# Sport-Specific Considerations in ACL Reconstruction: Diagnostic Evaluation and Graft Selection

**DOI:** 10.3390/diagnostics16040584

**Published:** 2026-02-15

**Authors:** Assala Abu Mukh, Giacomo Placella, Ki-Mo Jang

**Affiliations:** 1 Department of Orthopedic Surgery, Vita-Salute San Raffaele University, 20132 Milan, Italy; 2 Department of Orthopedic Surgery, Anam Hospital, Korea University College of Medicine, Seoul 02841, Republic of Korea; 3 Department of Sports Medical Center, Anam Hospital, Korea University College of Medicine, Seoul 02841, Republic of Korea

**Keywords:** ACL, sports, graft, revision, failure, instability, pivot

## Abstract

Knee biomechanical demands vary across different sports due to sport- and position-specific patterns of muscle recruitment. To return to performance, athletes must adequately restore knee kinematics to regain control over the same sport mechanics that led to the initial anterior cruciate ligament (ACL) injury. ACL graft selection should therefore minimize donor site morbidity and support sport-specific demands. This study aims to address the available evidence and guide surgical graft choice in athletes. A literature search of PubMed, MEDLINE, Scopus, and Web of Science (up to September 2025) assessed BPTB, hamstring, and quadriceps tendon autografts. Outcomes included revision, graft survival, return to sport, time to return, PROMs, anterior knee pain, donor site morbidity, and prognostic factors (age, sex). Sports were classified as pivoting, contact/collision, or endurance/non-pivoting. The results were synthesized narratively. In *pivoting* and *cutting sports*, bone–patellar tendon–bone (BPTB) autografts offer high survival rates but are associated with a high incidence of anterior knee pain, which is a substantial drawback in kneeling or flexion-intensive sports. Hamstring tendon (HT) grafts carry higher revision rates in female and younger patients, though they have low donor site morbidity that does not appear to affect long-term athletic performance. Quadriceps tendon (QT) grafts are emerging as a promising option for pivoting athletes. However, conflicting results indicate that the revision risk is comparable to that of HT grafts and possible long-standing extensor mechanism weakness. *Contact* and *collision sports* demonstrate similar trends, but kneeling and contact injuries are more common in this group. Thus, while prioritizing powerful hamstring strength, anterior knee pain symptoms should still be carefully considered. The diameter of the HT autograft should exceed 7.5 mm to ensure comparable revision outcomes with BPTB. QT grafts remain a limited-evidence attractive option. *Endurance* and *non-pivoting* athletes require fewer pivoting mechanics but rely heavily on muscle symmetry and repetitive motion. BPTB grafts are less suitable in this category due to alterations in sprint mechanics, muscle asymmetry, and repetitive patellofemoral joint loading. HT grafts provide favorable rates of return to sport, whereas evidence regarding QT graft use in non-pivoting athletes remains limited.

## 1. Introduction

Anterior cruciate ligament (ACL) tears are among the most frequent and consequential knee injuries, accounting for over 50% of all knee injuries sustained during athletic participation [1,2,3]. The injury mechanism often occurs after multiplanar loading patterns such as sudden torsional load, abrupt changes in direction, jump/landing sequences, pivoting during athletic activities, combined with valgus collapse and tibial translation [4,5,6]. Despite the substantial advances obtained in surgical techniques, rehabilitation protocols and injury prevention strategies, ACL injury still represents a major threat to athletic performance and career longevity due to its long-term social/physical impact.

ACL reconstruction (ACLR) has evolved beyond objective static stability; instead, the modern ACLR goal is to recreate functional knee kinematics capable of tolerating and adapting to sport-specific loads while preserving neuromuscular efficiency and minimizing donor site morbidity. The paradigm shift is particularly relevant in athletes who undergo ACLR and must withstand the same mechanical demands that mainly caused their initial injury. Failure to guarantee postoperative sport-specific functional demands not only compromises athletic return to sports (RTS) but also predisposes athletes to graft failure, contralateral knee injury and early retirement from competitive sports [7,8,9,10].

Pivot-dominant sports such as soccer, basketball and handball impose high rotational loads and rapid deceleration forces, whereas contact and collision sports introduce frequent direct trauma, kneeling positions and static pivoting. In contrast, endurance and non-pivoting sports rely heavily on cyclical loading patterns, muscle symmetry, and fatigue resistance rather than abrupt directional changes. These distinctions suggest that a “one size fits all” approach in ACL graft selection may be a biomechanically non-advantageous strategy and possibly inadequate for athletic populations [11]. Therefore, graft selection for ACL reconstruction should minimize donor site morbidity and support the functional demands specific to each sport [12,13].

Despite extensive research comparing graft types in ACL reconstruction, most studies evaluate heterogeneous athletic populations and focus primarily on graft survival and general functional scores. This approach overlooks the substantial variability in biomechanical demands across different sports and playing roles, as well as the sport-specific consequences of donor site morbidity. Consequently, current evidence provides limited guidance for tailoring graft selection to the functional requirements of athletes engaged in pivoting, contact, or endurance-based sports.

For the purpose of this review, athletic activities were broadly categorized into pivoting and cutting sports (e.g., soccer, basketball, handball, downhill skiing), contact and collision sports (e.g., wrestling, martial arts, American football), and endurance or non-pivoting sports (e.g., sprinting, long-distance running). To the best of our knowledge, few studies specifically address these sport-related demands in relation to graft selection. Therefore, this review aims to synthesize the available evidence and support surgical graft selection through a sport-specific, recent literature-based approach.

## 2. Materials and Methods

The current study was conducted based on the principles of evidence-based practice with a focus on the sport-specific characteristics and graft choice reported in athletes of various sport types. Research was conducted on PubMed, MEDLINE, Scopus and Web of Science up to September 2025. The objectives were to descriptively evaluate different outcomes of bone–patellar tendon–bone (BPTB) autograft, hamstring tendon (HT) autograft and quadriceps tendon (QT) autografts. Outcomes evaluated included revision and graft survival, return to sports (RTS), time to return to sports (TTRS), patient-reported outcome measures (PROMs), return to pre-injury level, anterior knee pain, donor site morbidity and patient-specific prognostic factors of age and sex across sports categories. Sports were categorized into three types: pivoting sports (soccer, basketball, handball, downhill skiing), contact and collision sports (wrestling, martial arts, American football), and endurance and non-pivoting sports (sprinters, long-distance runners). Search terms included MeSH terms of anterior cruciate ligament reconstruction, ACL graft, patellar tendon, hamstring tendon, return to sport, pivoting sports, contact sports, collision sports, endurance and non-pivoting sports. The references of the included studies were screened for additional resources. Due to the nature of the search, the results were synthesized narratively.

## 3. Diagnostic Evaluation

### 3.1. Physical Examination

Multiple tests can be used to assess ACL injury. Deriving from its anatomy, ACL has two main bundles, the anteromedial (AM) and posterolateral (PL) bundles. The AM bundle is mostly responsible for anterior tibial translation, whereas the PL bundle has major rotational control and some translational control in near extension.

The Lachman test and the anterior drawer (AD) tests are specific translational tests; therefore, they both predominantly assess the AM bundle [13]. The Lachman test is performed in slight knee flexion and is often positive in acute settings as its accuracy tends to “wane off” in chronic cases, whereas the AD test is performed at 90° of knee flexion and is less sensitive due to hamstring and secondary stabilizers’ activation at this anatomical position [14].

The PL bundle may be assessed through the pivot-shift test, which reproduces dynamic rotatory instability. By applying combined rotational and translational forces, the aim is to reproduce the “giving way” feeling which directly correlates it to the functional instability seen during athletic activities. The pivot-shift test exploits the anatomical function of the iliotibial tract (IT band) which functions as an extensor in low knee flexion degrees that “shifts” to being a flexor in higher degrees of flexion. In an ACL deficient knee, during knee flexion on an internally rotated tibia, when the IT band changes its function, there is a sliding of the tibial plateau compared with the femur which is felt by the examiner [15]. Whenever the slide is appreciated by others standing close to the examiner, the pivot-shift is graded as II, and when a clunk is heard, it is graded as III. Although the pivot-shift test is crucial for rotational instability assessment, it is heavily influenced by the examiner’s experience and subjective evaluation [16].

### 3.2. Objective Evaluation

In ACL injury, objective laxity may be assessed through instrumented tools such as arthrometer test, KT-1000 and KT-2000 that produce a millimetric side-to-side difference with mechanical stress radiography, between the affected and the contralateral limb. The unaffected knee serves as a standardized control for the injured limb, providing both a preoperative and a postoperative evaluation of static laxity [17]. Typically, a difference of 0–2 mm is considered normal, 3–5 mm indicates mild laxity, 6–10 mm moderate laxity, and >10 mm severe laxity. Although instrumented measurements provide precise quantitative data, they are inherently non-dynamic, and since exact thresholds may vary slightly depending on the device and study population, these instruments are complementary to clinical evaluation as they capture rotational and functional stability only partially. Therefore, instrumented assessments should be contextualized based on their clinical relevance from case to case [18].

A part of the objective evaluation assesses the range of motion, relevant in both the choice of graft and rehabilitation progress, as well as cases where arthrofibrosis may be suspected.

### 3.3. Imaging

Whenever there is a clinical suspicion of ACL injury, plain X-rays help exclude fractures in acute settings, and may indirectly indicate ACL involvement such as in cases of tibial eminence fractures in young patients and anterolateral ligament (ALL) avulsion fractures known as Segond fractures, and thus remain important for a comprehensive knee evaluation [19,20]. Although the definitive diagnosis is made arthroscopically, magnetic resonance imaging (MRI) is often mentioned as the gold standard for ACL injury diagnosis due to its high sensitivity and accuracy, which exceed 90% for complete ACL tears. Partial ACL tears can sometimes be missed on MRI and may be managed conservatively, highlighting the importance of careful clinical evaluation. MRI also evidences anatomical conformation, concomitant meniscal, cartilaginous and ligamentous injuries, all crucial for complete operative planning [21].

### 3.4. Special Considerations in Athletes

Assessment of pivot-shift, rotational instability, and MRI findings is essential for guiding surgical graft decisions, particularly in athletes engaged in high-demand, pivoting sports. Although rotational instability can impact daily activities, it is especially consequential in pivoting sports, where it increases the risk of secondary injuries and accelerates knee joint degeneration. Thus, in athletic patients, additional assessments relevant to neuromuscular control, strength testing, and proprioceptive evaluation are necessary [22,23]. These assessments heavily influence the athlete’s return to sports (RTS) timing and return to pre-injury sports level following an ACL reconstruction.

Based on the patient’s practiced sport and role, the patterns of muscle activation may differ significantly; therefore, an evaluation of sport-specific muscle performance comparing the affected limb with the contralateral side prior and following surgery becomes mandatory [24]. Muscle performance assessment may help reduce donor site morbidity by sparing case-specific grafts that are predominantly used in each sport, by delaying game or by intensifying rehabilitation programs on sport-specific exercises, thus permitting postoperative RTS to the desired level [25].

Although isokinetic and isoinertial dynamometers should be used for a comprehensive muscle assessment following an ACL injury, the mechanical designs between the available machines vary considerably, importantly, these devices do not truly isolate individual muscles but rather assess net torque produced by muscle groups acting on the knee. This limitation is particularly relevant in the athletic population, as results may reflect a specific altered neuro-motor control rather than true standalone muscle weakness [26]. Therefore, machine-based data must be interpreted within a broader functional context that integrates neuromuscular control, proprioception, and sport-specific performance measures, holding the contralateral limb as a valuable monitoring resource to guide recovery and RTS [27].

## 4. Graft Options

Graft options for ACL reconstruction primarily include autografts, allografts and synthetic grafts. Robust clinical evidence consistently demonstrates that allograft use is associated with inferior outcomes compared with autografts. Specifically, compared with autografts, allografts have been reported to lead to two to four times higher rates of graft failure, increased knee laxity, and lower rates of return to sport, particularly in young and active populations, the main recipients of ACL reconstruction [28].

Although emerging options for autografts including the peroneus longus tendon (PL) and rectus femoris tendon are gaining popularity, there is limited evidence regarding their long-term outcomes and these will not be discussed in this article. Consequently, the current recommendation is to use autografts such as bone–patellar tendon–bone (BPTB), hamstring tendon (HT), or quadriceps tendon (QT) for primary ACL reconstruction, particularly in high-risk patients. Allografts may be considered for select older, less active individuals or in cases of multiple knee injuries [28,29,30,31]. The overall characteristics of autografts are summarized in Table 1.

### 4.1. Pivoting and Cutting Sports

This category includes team sports like soccer and basketball, where athletes are exposed to high-demand forces, including pivoting, cutting maneuvers, and direct contact. The mechanisms of injury often vary by field position and role. Soccer wingers frequently sustain non-contact injuries during explosive cutting or landing tasks, whereas midfielders and defenders are more prone to contact-related injuries [32]. Regardless of injury mechanism, players are generally expected to return to their original positions following ACL reconstruction, making restoration of optimal knee stability essential in regaining pre-injury performance levels.

Additional factors like sex and age play heavily affect injury risk and prognosis. Female athletes are known to have a higher risk of ACL injury, graft re-tears, and contralateral knee injuries compared with their male counterparts. Consequently, when these factors intersect, careful consideration of graft selection is crucial to achieving a robust reconstruction strategy and optimizing athletic outcomes [32,33].

#### 4.1.1. BPTB Versus HT

When addressing ACL reconstruction in pivoting sports, evidence suggests that both HT and BPTB autografts are valid options; however, subtle differences should be considered based on the desired outcome. Although there is general agreement that BPTB autografts may provide better outcomes for athletes in pivoting sports, the athlete’s specific expectations should be clarified before determining the surgical strategy. Is the primary goal RTS, time to return to sport (TTRS), reducing revision risk, or return to pre-injury performance levels?

Revisions: Age appears to be a significant revision-modifying factor as reported by Busch et al. Their study found that HT graft failure occurred at a younger mean age (14.9 years) compared with BPTB graft failure (15.9 years) and that revision rates were significantly higher in the HT group [34] reaching nearly fivefold in athletes younger than 18 [35]. On the other hand, a study by Salem et al. mentions that when allograft-augmented HT grafts were excluded, there was no significant difference in revision rates between the HT and BPTB groups, while donor site morbidity remained significantly higher in the BPTB group [36,37]. This tendency was confirmed by a meta-analysis where re-rupture rates of BPTB (2.2%) were statistically comparable with HT (2.5%) [38,39]. Long-term outcomes indicate a significantly higher risk of revision with HT (93%) ACL reconstruction compared with BPTB (96%) at 12 years from index surgery, particularly in younger soccer, handball, and alpine skiing athletes [40,41]. However, these findings were contradicted by other studies involving netball, rugby, and soccer athletes [35,41]. Studies report that 18-year-old males present a 44% risk of re-injury [42], while female volleyball athletes are more prone to ACL injury due to landing mechanics predisposing to valgus–external rotation [43]. Overall, nearly one-third of young athletes participating in pivoting sports experience a secondary ACL injury, emphasizing the intrinsically high risk in this population [44,45].

RTS and return to pre-injury level: Studies indicate no significant difference in RTS rates with BPTB autografts (81.0%) compared with HT autografts (70.6%) as well as return to pre-injury levels between BPTB (48.9%) and HT (50.0%) groups [40]. However, HT autografts demonstrated superior medium-term International Knee Documentation Committee (IKDC) scores (*p* = 0.017) and significantly reduced long-term anterior knee pain symptoms compared with BPTB autografts (*p* = 0.045), both important for early rehabilitation, RTS, and long-term athletic performance [46,47].

Stability and strength: Compared with HT autografts, BPTB autografts provided significantly greater stability in pivoting female athletes younger than 20 years, as measured by side-to-side difference (STSD) on radiographs at 5 and 12 months postoperatively. Interestingly, the same study found no significant difference in quadriceps muscle strength but significantly better hamstring strength in the BPTB group [41].

Morbidity: The most reported donor site morbidity is consistently associated with BPTB autograft use and includes anterior knee pain and, rarely, patellar fractures. The incidence of anterior knee pain is a significant drawback that often limits athletic performance. It has been reported to reach as high as 50.5% of cases with BPTB autografts compared with 2% with HT ACL reconstructions (*p* = 0.04) [36,37,38]. Kneeling pain should be carefully evaluated based on the athlete’s sport, such as volleyball and skiing. These findings suggest that although both BPTB and HT autografts are viable options they each exhibit superiority in different outcomes.

#### 4.1.2. QT Versus HT and BPTB

QT autograft use has recently gained popularity as an alternative to BPTB and HT autografts in pivoting and cutting sports.

Re-rupture, patient-reported outcome measures (PROMs), RTS, and TTRS: A comparative study evaluated soccer, football, lacrosse, and basketball athletes’ PROMs, RTS, TTRS, and re-tear rates between QT and BPTB autografts reporting no significant differences in IKDC and Lysholm scores, TTRS (7.1–7.6 months), or RTS rates (90.6% with QT vs. 86.1% with BPTB; *p* = 0.82) [48]. Although some sport-nonspecific studies reported better rotational control and lower re-rupture rates with QT autografts (odds ratio 0.46) compared with HT autografts, STSD and PROMs were still comparable between groups [49].

QT autografts also demonstrated fewer donor site morbidities and kneecap-related symptoms compared with BPTB autografts (odds ratio 0.14–0.16) [50]. Nevertheless, a recent study by Zegzdryn et al. reported a higher risk of revision in QT autograft recipients compared with BPTB recipients, while maintaining similar failure rates to the HT group, highlighting the need for long-term research before establishing the definitive role of QT autografts in athletic populations [51].

Strength: An in vivo kinematic study indicated that HT autograft use may result in increased anterior tibial translation and decreased flexion strength compared with BPTB and QT autografts, and a similar extensor deficit in patients receiving BPTB and QT autografts that did not significantly affect PROMs [31]. QT harvest leads to an approximate reduction of 34% in quadriceps strength, compared with 25% reduction in patellar tendon tensile strength following BPTB harvest. Both potentially contribute to long-term strength and functional knee deficits compared with HT autografts [52]. Consequently, the choice between QT and HT should involve a careful sport- and role-specific assessment, considering whether it is knee flexion or extension that plays a secondary role in the athlete’s performance demands.

### 4.2. Contact and Collision Sports

Graft survival and diameter: A study of wrestlers reported an 80% return to competitive wrestling after ACL reconstruction, with 14% experiencing a secondary ACL failure. At 15 years post index surgery, BPTB graft survival (90.4%) was significantly higher than HT autograft survival (76.3%) (*p* = 0.03). However, when HT autografts with a diameter smaller than 7.5 mm were excluded, failure rates were comparable between HT and all BPTB graft sizes [53]. To achieve a larger graft diameter, Jeffers et al. reported good outcomes with quadrupled HT autografts in football athletes, with an RTS rate of 85% and a re-injury rate of 6.9% [54].

RTS: Data regarding graft selection in contact and collision sports remain limited. A study of martial arts athletes reported comparable RTS rates among BPTB, HT, and QT autografts (*p* = 0.47) [55]. Takazawa et al. reported favorable outcomes in rugby players receiving augmented HT autografts, with RTS rates of 90–92%, longer time to failure, and lower failure rates in patients older than 20 years compared with younger patients (5% vs. 23%; *p* = 0.006) [56] where the majority of rugby players returned to sports within 12 months when BPTB was used [57,58].

In National Football League athletes, offensive and defensive linemen demonstrated a 64.3% RTS rate, and those who returned did so at high levels without a significant difference in performance or career length [59]. In contrast, some studies report lower RTS rates (50%) in football players [60], while others indicate comparable RTS (95%) and return to pre-injury levels (90%) among football and rugby elite athletes. Interestingly, rugby players demonstrated a shorter TTRS compared with football players (9.6 vs. 10.6 months; *p* = 0.027) and football players were more likely to receive BPTB autografts than rugby athletes [61].

Although some papers logically advise against the use of HT autografts in sports requiring sprinting and substantial hamstring strength [62], given the current state of evidence, it may be premature to definitively recommend against a specific autograft type in the contact and collision category, particularly considering the survival and stability of ACL reconstructions with adjunct anterolateral ligament (ALL).

### 4.3. Endurance and Non-Pivoting Sports

This category of athletes is supported by the least amount of research evidence as the literature primarily addresses HT autograft use in this population.

Return to running: One study focusing on a structured rehabilitation program reported that 97% of athletes receiving HT autografts successfully completed a short-term return-to-running program, and IKDC scores above 64 predicted program completion [63,64].

Biomechanics and morbidity: Regardless of graft, studies report that running biomechanics remain altered 12 months after the index surgery, demonstrating substantial deficits compared with the pre-injury state that persist beyond the RTS period [65]. Although BPTB autografts have been suggested to protect against secondary injury by mitigating flexion–extension asymmetry, HT autograft harvest alters hamstring muscle activation during running as well [66]. These neuromuscular deficits theoretically increase the risk of hamstring strain injuries, particularly near the end of the swing phase of running and sprinting. However, these theoretical deficits do not appear to delay the timing of RTS progression [67,68], emphasizing the importance of a dedicated rehabilitation program for runners, which differs from that of athletes in pivoting sports [69]. Guglielmetti et al. compared outcomes of BPTB and HT autografts and found that, although knee pain was significantly more common in the BPTB group, loss of kicking power, sprint start performance, and tendinitis rates were comparable between groups, suggesting HT autografts to be a favorable option for long-distance runners. However, the study was conducted primarily on soccer athletes rather than exclusively on non-pivoting athletes [70].

The growing interest in QT autografts is supported by their low complication rates, favorable medium-term outcomes, and preservation of hamstring muscle function and swing mechanics. These findings suggest QT autografts may represent a promising option for high-volume runners. Nonetheless, further high-quality, long-term studies are needed to confirm the advantages of QT autografts in this population [71,72,73].

## 5. Discussion

When considering RTS, TTRS, and PROMs, all autograft options appear to provide comparable results. Therefore, revision rates and donor site morbidities become the key outcome parameters to balance when selecting the graft and should integrate patient-specific factors with an understanding of sport-specific biomechanical demands [48,57].

In pivoting and cutting sports, abrupt directional changes, deceleration, and landing represent high-risk mechanics. Thus, regardless of graft choice, sport-specific injury prevention programs should target these movements. BPTB autografts appear to offer both high survival rates and high complication rates, making them a suitable option for young female pivoters [32,33]. However, the relatively high incidence of anterior knee pain is a considerable drawback, particularly for sports that require kneeling or deep flexion. Although HT autografts are associated with higher revision rates in athletes younger than 20 years, their use permits early rehabilitation and RTS while maintaining low donor site morbidity without compromising long-term athletic performance. QT autografts represent an emerging option for pivoting sports; however, current evidence is conflicting, suggesting possible long-standing extensor mechanism weakness and a revision risk comparable to that of HT autografts at two years postoperatively. These findings call for cautious use of QT autografts until additional high-quality evidence becomes available [51].

Despite contact and collision sports showing outcomes like those in pivoting sports, kneeling, stationary pivoting actions, and contact injuries are more frequent and place the ACL at greater risk of primary and secondary injury. Since athletes who return to sport often do so at the same competitive level, donor site morbidity associated with BPTB autografts should be carefully considered to permit return to pre-injury levels. Wrestlers, who require substantial hamstring strength, should be considered carefully for HT autografts, and when used, HT graft diameter greater than 7.5 mm is desirable to ensure revision outcomes comparable to those of BPTB autografts [53]. QT autografts represent an attractive option for this category; however, given the need to balance durability and morbidity while ensuring favorable RTS rates, current evidence remains heterogeneous and limited.

Endurance and non-pivoting athletes face fewer pivoting demands but rely heavily on muscle symmetry and repetitive motion. Consequently, BPTB autografts appear less suitable in this category as they increase repetitive patellofemoral joint stress and alter both sprint mechanism and muscle asymmetry in long-distance runners. HT autografts are generally considered a good option for endurance athletes for a successful RTS, and theoretical concerns regarding the risk of hamstring strain are successfully addressed through dedicated prevention programs [65,66,69]. Evidence regarding the use of QT autografts in non-pivoting athletes remains scarce and controversial, largely due to concerns about potential muscle asymmetry and the lack of robust long-term outcome data [51].

**Figure 1:**
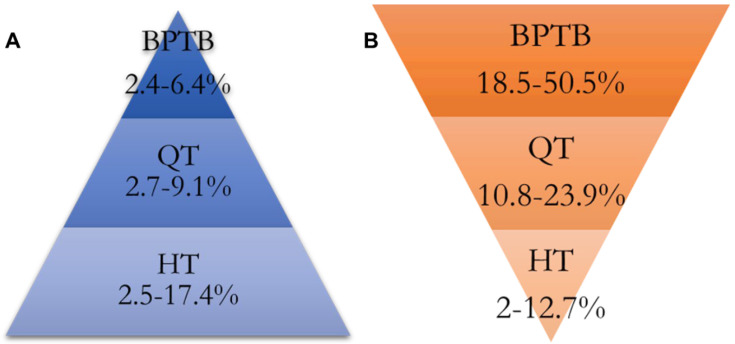
Reported failure rates (**A**) and prevalence of anterior knee pain (**B**) based on the graft choice. BPTB-bone-patellar tendon-bone, QT-quadriceps tendon, HT-hamstring tendon.

Considering the general literature, although reported failure rates vary, they typically range from approximately 2.4 to 6.4% for BPTB autografts, 2.7–9.1% for QT autografts, and 2.5–17.4% for HT autografts (Figure 1A) [74]. Reported rates of anterior knee pain (Figure 1B) are frequently variable, ranging from 18.5 to 50.5% for BPTB, 10.8–23.9% for QT, and 2–12.7% for HT autografts. This variability is likely due to differences in study populations and surgical heterogeneity, such as the use of soft-tissue QT versus bone-plug QT grafts [75,76,77,78,79].

Finally, a meta-analysis by Haybäck et al. calculated the yearly incidence of graft failure and found no statistically significant differences among the three graft types [80]. Therefore, across all sports categories, graft choice should be tailored to sport-specific and patient-specific factors. Given the consistently higher revision rates reported in high-risk populations, adjunctive procedures such as ALL reconstruction may be considered to enhance rotational stability while balancing the morbidity trade-offs associated with BPTB grafts; ALL reconstruction may play a role in reducing revision risk and mitigating anterior knee pain symptoms deriving from BPTB harvest [81,82,83,84]. Therefore, future research should investigate the benefits of adjunctive procedures by comparing outcomes of BPTB, HT, and QT autografts with and without ALL reconstruction, particularly in high-risk populations.

This review has several limitations. First, although emerging autograft options such as the peroneus longus and rectus femoris tendons have been reported in recent research, current evidence is limited by small sample sizes, short follow-up, and a lack of sport-specific outcome measures, precluding meaningful conclusions regarding their implications in competitive athletes [85,86,87]. For this reason, the present review focuses on grafts supported by more robust evidence in athletic populations, thereby allowing more reliable interpretation and clinical applicability. In addition, the literature on graft selection in athletes is heterogeneous with respect to study design, surgical technique, rehabilitation protocols, and definitions of return to sport, with many studies failing to stratify outcomes by sport type or performance level. To mitigate this limitation, a sport-based conceptual framework was adopted to synthesize existing data in a clinically interpretable manner.

Finally, although categorizing sports into pivoting, contact, and endurance-based activities inevitably simplifies the spectrum of biomechanical demands, this approach provides a pragmatic structure to integrate biomechanical principles with clinical outcomes. Importantly, by emphasizing donor site morbidity in relation to sport-specific functional demands, this review addresses common surgeon concerns regarding potential performance-limiting morbidities associated with graft choice in different sports. Despite these limitations, this study offers a focused, sport-specific synthesis of contemporary evidence and delivers clinically relevant guidance to support individualized graft selection while identifying key areas for future athlete-centered research.

## 6. Conclusions

Graft selection for ACL reconstruction in athletes should be individualized according to sport-specific biomechanical demands rather than based on a uniform strategy. While BPTB, HT, and QT autografts yield comparable RTS rates and PROMs, differences in revision risk and donor site morbidity remain clinically relevant. In pivoting and cutting sports, BPTB grafts provide reliable stability but are frequently associated with anterior knee pain, whereas HT grafts offer lower donor site morbidity with a higher susceptibility to revision in younger and high-risk athletes. In contact and collision sports, graft durability must be balanced against functional morbidity, making adequately sized HT or QT grafts reasonable alternatives. For endurance and non-pivoting athletes, HT grafts appear most suitable due to preserved muscle symmetry and reduced patellofemoral loading. In high-risk athletic populations, adjunctive ALL reconstruction may represent a complementary strategy to enhance rotational stability and potentially reduce revision risk when using HT grafts, while maintaining the advantage of low donor site morbidity. Overall, graft choice should be guided by an integrated assessment of sport demands, athlete characteristics, and acceptable morbidity to optimize long-term athletic outcomes.

## Figures and Tables

**Table 1:** Summary of autograft performance.

Graft	Revision Risk	Donor Site Morbidity	PROMs, RTS, TTRS	Muscle Morbidity	Contralateral Injury Risk
BPTB	Low	High	Comparable	Extension	High
HT	High	Low	Comparable	Flexion	Low
QT	Mixed/Contradictory	Mixed/Contradictory	Comparable	Extension	Low

BPTB, bone–patellar tendon–bone autograft; HT, hamstring tendon autograft; QT, quadriceps tendon autograft; PROMs, patient-reported outcome measures; RTS, return to sport; TTRS, time to return to sport.

## Data Availability

No new data were created or analyzed in this study. Data sharing is not applicable to this article.

## Abbreviations

The following abbreviations are used in this manuscript:

ACL	Anterior cruciate ligament
ACLR	Anterior cruciate ligament reconstruction
AD	Anterior drawer
ALL	Anterolateral ligament
AM	Anteromedial
BPTB	Bone–patellar tendon–bone
HT	Hamstring tendon
IKDC	International Knee Documentation Committee
IT	Iliotibial (tract)
KT-1000	Knee arthrometer (KT-1000)
KT-2000	Knee arthrometer (KT-2000)
MRI	Magnetic resonance imaging
PL	Posterolateral
PROMs	Patient-reported outcome measures
QT	Quadriceps tendon
RTS	Return to sport
STSD	Side-to-side difference
TTRS	Time to return to sport
